# Importance of sessile serrated lesions in a patient with familial adenomatous polyposis

**DOI:** 10.1007/s12328-021-01498-0

**Published:** 2021-08-29

**Authors:** Motoki Watanabe, Hideki Ishikawa, Shingo Ishiguro, Michihiro Mutoh

**Affiliations:** 1 Department of Molecular-Targeting Prevention, Kyoto Prefectural University of Medicine, Kawaramachi-Hirokoji, Kamigyo-ku, Kyoto, 602-8566 Japan; 2 Pathology and Cytology Laboratories Japan, 1-34-5 Koenji-Minami, Suginami-ku, Tokyo, 166-003 Japan

**Keywords:** Familial adenomatous polyposis, Sessile serrated lesion, Endoscopic polypectomy

## Abstract

A 28-year-old male visited hospital because his mother had been diagnosed with familial adenomatous polyposis (FAP) with a pathological variant of the *APC* gene. Total colonoscopy showed that he has more than 100 polyps distributed throughout the colorectum, and the *APC* gene variant was also detected. After he was diagnosed with FAP, he received information that surgery was currently the only way to prevent the development of colorectal cancer. However, he firmly declined to undergo surgical procedures and decided to have strict follow-up with frequent endoscopic polypectomy to prevent the development of colorectal cancer. At the first endoscopy, polypectomy was performed on 52 polyps. Histological analysis of the dissected polyps showed that they were all adenomas, but adenocarcinoma was not detected. The second endoscopic polypectomy was performed after 4 months later. We found a pale 20 mm wide flat, elevated type polyp in the ascending colon with an adherent mucus cap that was resistant to washing off. After endoscopic mucosal resection, histological analysis revealed that there were two lesions in the polyps, a sessile serrated lesion (SSL) and SSL with dysplasia. SSL is a high-risk lesion for colorectal cancer, but it was reported to be rare in patients with FAP, and the existence of SSL suggested another carcinogenesis pathway in patients with FAP in addition to the adenoma-carcinoma sequence. Our report may be significant not only in consideration of the pathogenesis of FAP but also useful to raise awareness of SSL for clinicians who perform endoscopic polypectomy to prevent the development of colorectal cancer in patients with FAP.

## Introduction

Familial adenomatous polyposis (FAP) carries a high-risk of colorectal cancer, characteristically manifesting as hundreds of adenomatous polyps in the colorectum. This syndrome is caused by germline mutations of the *APC* gene, a tumor-suppressor gene, located on chromosome 5q21 [1]. The reported prevalence of FAP is 3–10/100,000, but colorectal cancer will develop around the fourth decade of life, without prompt treatment at younger ages [1]. To date, extirpating the entire colorectal mucosa by surgery is only way to prevent colorectal cancer development. Recently, we reported retrospective data that demonstrated the usefulness of regular colonoscopy and polypectomy without surgical procedures to prevent the development of colorectal cancer in the patients with FAP [2]. In this method, the colorectal polyp size and its surface characteristics may be one of the landmarks of specimens with suspected cancers during the resection of colorectal polyps.

This novel method, however, needs more information about endoscopic findings for general clinical use. Here, we report a case of a sessile serrated lesion (SSL), a high-risk lesion for colorectal cancer, with FAP in a young male who firmly declined to undergo surgical procedures and decided to have strict follow-up with frequent endoscopic polypectomy to prevent the development of colorectal cancer.

## Case report

A 28-year-old male visited our hospital because his mother has been diagnosed with FAP with a pathological variant of the *APC* gene. The *APC* gene in his mother was screened using the protein truncation test (PTT), and abnormal bands were found in segments of the *APC* gene (codons 658–1283).

**Fig. 1:**
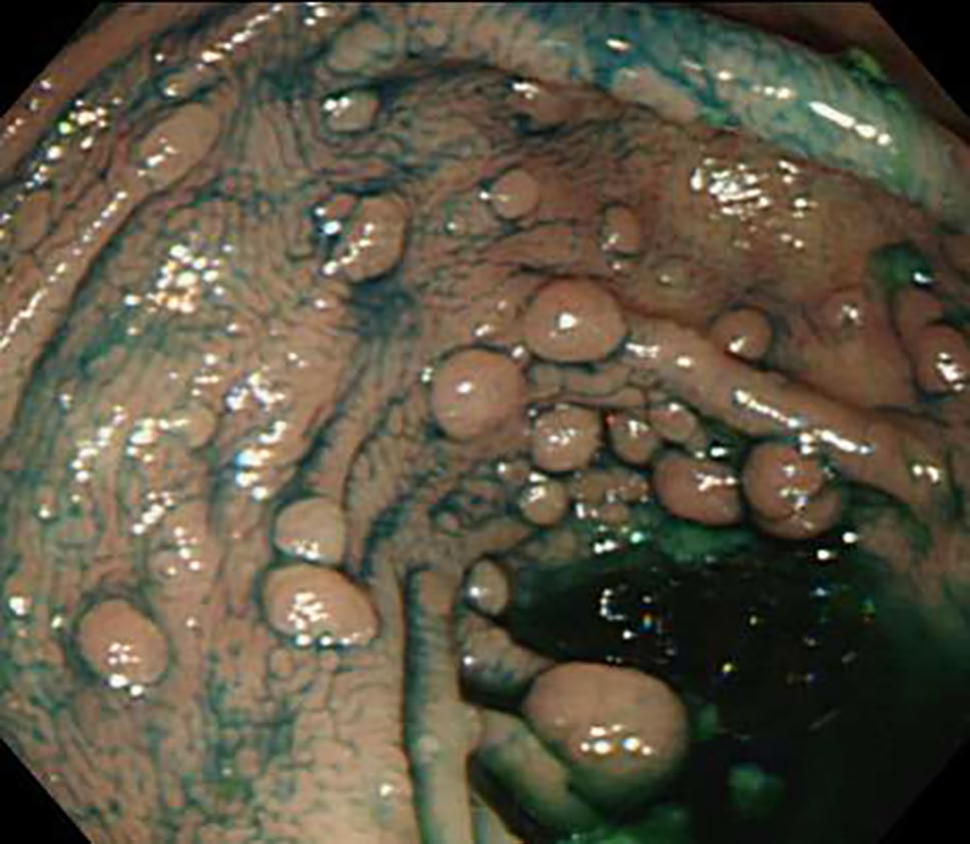
Endoscopic appearance in the colon with indigo carmine dye spraying. Colonoscopy demonstrated the presence of more than 100 polyps, each up to 20 mm in size, distributed throughout the colorectum

Total colonoscopy showed that he had more than 100 polyps distributed throughout the colorectum (Fig. 1). Further examination revealed that he also possessed the pathological variant of the *APC* gene (Exon15 c.2751del (p.Asp917Glufs*38)). After he was diagnosed with FAP, he received information that surgery was currently the only way to prevent the development of colorectal cancer. However, he firmly declined to undergo surgical procedures and decided to have strict follow-up with frequent endoscopic polypectomy to prevent the development of colorectal cancer. At the first endoscopic examination after diagnosis, en bloc resection was performed on the maximum diameter of polyps larger than 20 mm, and in total 52 large polyps were resected. In histological examination, all polyps were tubular adenomas (~ high-grade dysplasia) and not adenocarcinomas.

**Fig. 2:**
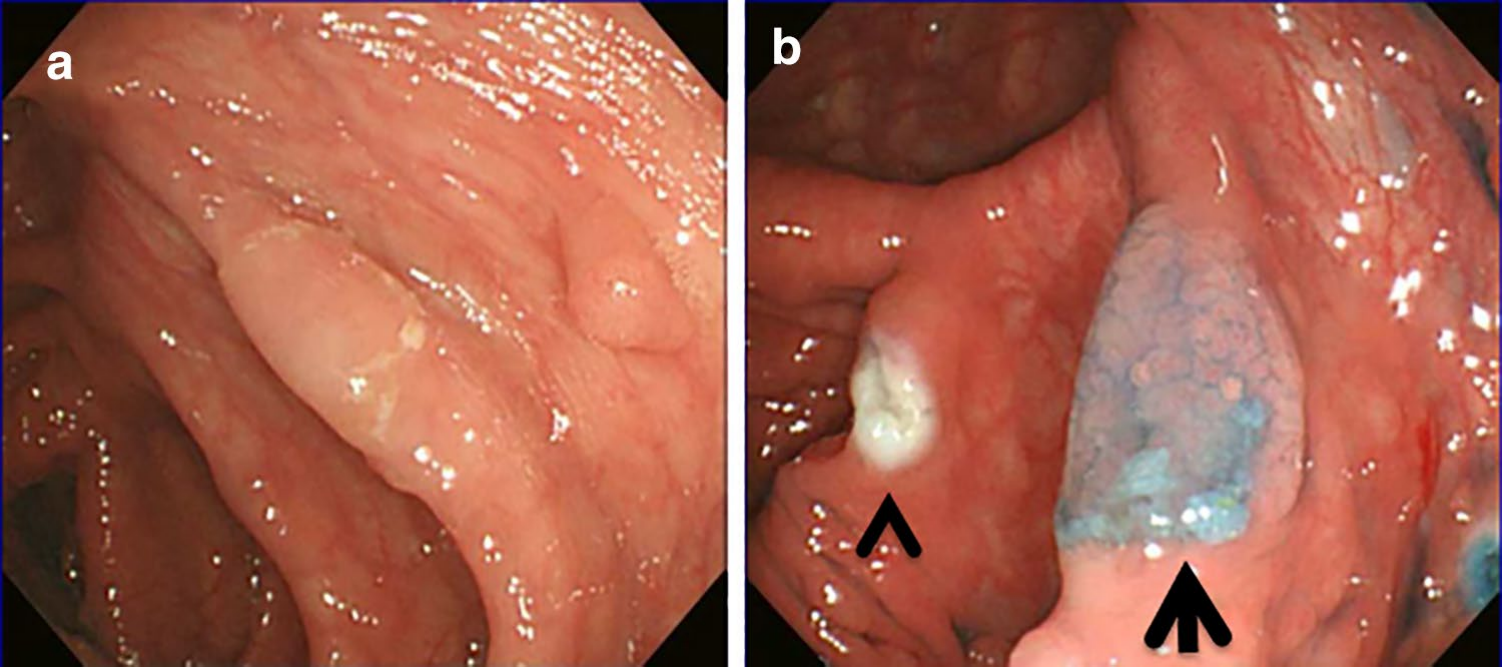
Endoscopic finding of a serrated lesion. **a** A sessile serrated lesion with adherent mucus cap that was difficult to wash off. **b** The lesion (arrow) after spraying with indigo carmine. The arrowhead shows the scar of polyp resection

**Fig. 3:**
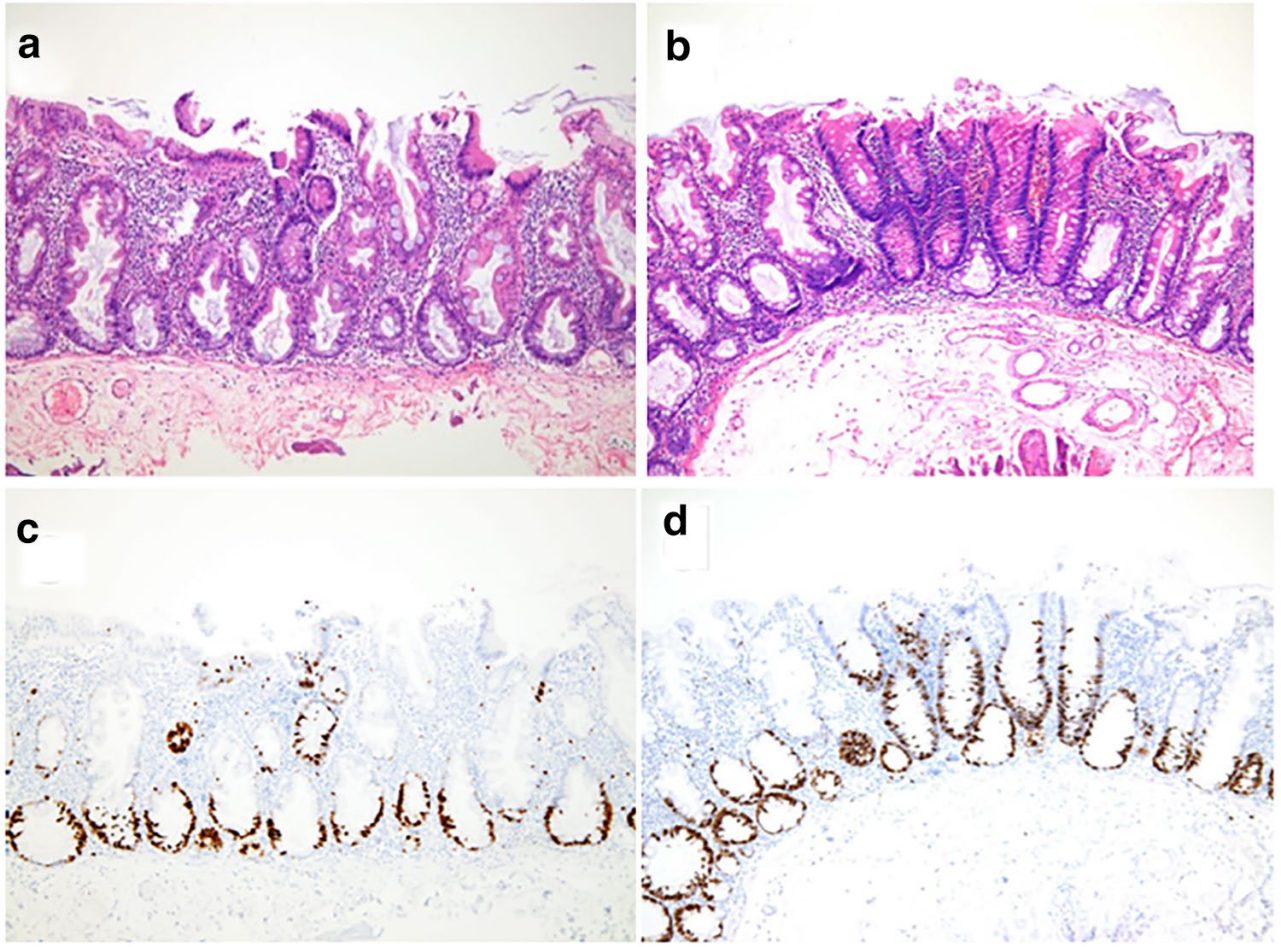
Histopathology of the polyps. **a** HE staining of the sessile serrated lesion (SSL). **b** HE staining of the sessile serrated lesion (SSL) with dysplasia. **c** Immunohistochemical staining of successive section to **a** with an anti-Ki67 antibody. **d** Immunohistochemical staining of successive section to **b** with an anti-Ki67 antibody

The second endoscopic polypectomy was performed 4 months later. We found a flat elevated type polyp, 20 mm in size, located in the ascending colon (Fig. 2a). It was on a fold edge and disrupted the vascular pattern of the normal colonic mucosa. Spraying with indigo carmine revealed that it had an irregular cerebriform surface and was paler than the background mucosa with an adherent mucus cap that was not washable (Fig. 2b). After endoscopic mucosal resection, histological analysis revealed that there were two lesions in the polyp, SSL (Fig. 3a) and SSL with dysplasia (Fig. 3b). SSL showed characteristics of saw-tooth protrusions of the epithelium into the glandular lumen comparable with hyperplastic polyps but focally with a more complex architecture, and T-shaped or L-shaped glands and dilated glands at the base of the lesion just above the muscularis mucosae. Goblet cells reached the base of the lesion. Ki67 staining showed abnormal cell growth in the tissue of SSL and SSL with dysplasia (Fig. 3c and 3d).

## Discussion

Sections taken from the colorectal mucosa in patients with FAP often contain adenomas and microscopic adenomas, so-called aberrant crypt foci. Conventional adenomas are precursors of the majority of colorectal adenocarcinomas that harbor *APC* mutations. On the other hand, SSL is the most common precursor for sporadic microsatellite unstable-colorectal cancer. Thus, it is believed that SSL should be removed entirely, as with traditional serrated adenoma (TSA) which has also premalignant potential.

SSLs in patients with FAP have been previously considered to be rare, and we found four articles reporting SSL/serrated adenoma (SA) and TSA with FAP as far as we searched in PubMed. The first report was presented by Matsumoto et al. in 2002 [3]. They found SAs in 3 of 11 subjects examined. Three lesions (3.7%) were from three unrelated subjects. However, they suggested that the true incidence of SA in patients with FAP is probably lower (2.2%). They found SA uniformly in the rectum and these were small in size. These patients had less than 100 macroscopic polyps. Pedigrees with SA had truncated germline *APC* variants at codon 161, 332, or 1556, whereas in the other pedigrees variants were found between codons 554 and 1324.

The second report presented by Sawyer et al. described 8 SAs from patients with FAP [4]. Interesting, they showed that Wnt pathway abnormalities including the mutations of *APC* or *CTNNB1* (coding β-catenin) were also found in 6 other SAs from non-FAP patients, suggesting that the pathogenesis of a subset of SAs may be involved in *APC* and other Wnt pathway abnormalities.

The third report regarding SA with FAP patients was presented by Lee et al. [5], and they reported a very rare mutation of *BRAF*, with a triplet deletion of the coding nucleotides 1799 to 1801, in SA from a patient with FAP. Of note, the patient had a typical FAP course with germline variants of the *APC* gene and early occurrence of colon cancer combined with multiple adenomatous polyps.

The fourth report presented by Okamura et al. clarified the molecular features of 37 TSAs from 21 FAP patients using next-generation sequencing and Sanger sequencing [6]. They showed *KRAS* and *BRAF* V600E mutations were observed in 18 (49%) and 14 (38%) FAP-associated TSAs, respectively, suggesting that the pathogenesis of FAP-associated TSAs is similar to that of sporadic TSAs.

In our patient, the patient had more than a hundred polyps, and a truncating germline *APC* deletion was found at codon 2751. The adenoma was located in the ascending colon and was large in size. Thus, our case was not consistent with some reports that suggested SSL may be associated with less impaired function of the *APC* gene [7, 8] and may be characteristic of an attenuated FAP course [3].

In conclusion, we report the first identified case of SSL with dysplasia in the ascending colon of the FAP patient. This report is significant for considering the pathogenesis of FAP; however, the role of SSL in carcinogenesis in patients with FAP still remains unclear and accumulation of more cases with genetic analysis are needed. Furthermore, this can be helpful for alerting endoscopists who need to reduce the risks of overlooking a high-risk lesion for colorectal cancer, such as SSL during long-term follow-up of patients with FAP by endoscopy. Accumulation of information regarding high-risk lesions detected by endoscopy and the improvement of devices may allow the use of frequent endoscopic polypectomy to prevent patients with FAP from developing colorectal cancer in the future.
